# Beamforming for the Cooperative Non-Orthogonal Multiple Access Transmission with Full-Duplex Relaying with Application to Security Attack

**DOI:** 10.3390/s25041172

**Published:** 2025-02-14

**Authors:** Duckdong Hwang, Sung Sik Nam, Janghoon Yang, Hyoung-Kyu Song

**Affiliations:** 1 Department of Information and Communication Engineering, Sejong University, Seoul 05006, Republic of Korea; duckdonh@yahoo.com; 2 Department of Electronic Engineering, Gachon University, Seongnam 13120, Republic of Korea; ssnam@gachon.ac.kr; 3 Computer Science, Pennsylvania State University, Abington, PA 19104, USA; 4 Department of Information and Communication Engineering and Convergence Engineering for Intelligent Drone, Sejong University, Seoul 05006, Republic of Korea; songhk@sejong.ac.kr

**Keywords:** full-duplex relay, multiple antenna beamforming, non-orthogonal multiple access, security attack

## Abstract

We investigate the cooperative non-orthogonal multiple access (CNOMA) transmission through a full-duplex (FD) decode-and-forward (DaF) mode relay and propose two sub-optimal beamforming schemes for this CNOMA FD relay channel. For the optimization metric, we use the end-to-end information rate based on the mutual information from information theory. In addition to the pure CNOMA relay channel, the proposed beamforming schemes are applied to the security attack case as well, where an unauthorized eavesdropper tries to overhear the CNOMA transmission. The FD operation incurs the self-interference (SI) at the relay and the DaF mode along with CNOMA transmission forces the weakest link among the links toward three involved nodes to determine the end-to-end throughput. These facts lay the foundation for the designing and optimization of the beamforming vectors at the access point (AP) and at the relay. The first proposed sub-optimal optimization algorithm for the beamformer relies on the quadratically constrained quadratic problem (QCQP) in its central part, and this OCQP is iteratively applied with different interference level values at the near CNOMA user as the constraint term until some conditions for the design objectives are met. In addition to the first algorithm, a zero-forcing-based beamforming algorithm is proposed for a reference scheme. The proposed two algorithms are slightly modified to address the security-attacked CNOMA FD relay channel when a illegal user overhears the legitimate transmission. Simulation results are presented to advocate for the efficiency of the proposed algorithms for the CNOMA channel both with and without a security attack from an eavesdropper.

## 1. Introduction

Recent research on the non-orthogonal multiple access (NOMA) technologies [1] is driven by the huge numbers of small devices in addition to smart phones nowadays, which will be served in the coming sixth generation (6G) wireless systems. The 6G systems should accommodate these devices without much additional wireless resources or noticeable latency increase. The power domain NOMA in [2,3,4] and the sparse code multiple access [5,6] are attractive since they do not require additional spectral resources or a new multiple access mechanism in implementing the NOMA principle. The signals in the power domain NOMA toward multiple users are superimposed into a single spectral resource, which is contrasting to the orthogonal multiple access (OMA). The signals of OMA are allocated onto different orthogonal resources and hence the received signals of users in the OMA schemes are almost free from the inter-user interference (IUI). However, the IUI does matter in NOMA schemes, and the successive interference cancellation (SIC) is adopted to combat the IUI. To improve the performance of NOMA systems, several different approaches for resource allocation have been proposed [7,8,9]. The feasibility of NOMA systems over the millimeter wave band has also been studied [10,11].

Multiple antennas technologies [3,4,12,13,14,15,16,17,18,19], exploiting the spatial dimensions, can improve various performance metrics of the NOMA system including the secrecy capacity of the NOMA network [20]. Both the access point (AP) and the user terminals (UTs) are equipped with multiple antennas in the multiple-input multiple-output (MIMO) NOMA case. UTs are grouped into clusters, where the UTs in each cluster form independent NOMA channels that are distinct from those in other clusters [13,14,15]. In the multiple-input single-output (MISO) system, only the AP is equipped with multiple antennas, and the clustering of the MIMO case is not available. Only single-channel NOMA UTs [12,21,22] can be served by the beamforming at the AP in this case. The authors in [21] studied the optimal beamforming for this MISO-NOMA system. When UTs assist in NOMA transmission by relaying the AP signal to other UTs—either in half-duplex (HD) mode or full-duplex (FD) mode—the system is referred to as a cooperative NOMA system [23,24,25,26,27,28] and the additional spectral resource in the process returns improved spectral efficiency. The cooperative NOMA is helpful when some UTs are near the AP and they can help the UTs far away from the AP. The FD mode cooperative NOMA [23,24] is attractive since it does not need the additional spectral resource and thus is spectrally efficient. The two signal transmissions (at the AP and at relaying UTs) are performed in the same spectral resource, which makes the handling of self-interference (SI) at the FD relaying UTs a challenging task.

We, in this article, consider the beamformer optimization for the cooperative NOMA system with FD decode-and-forward (DaF) relaying, where multiple antennas are adopted at the AP and the FD relay (FDR). Two NOMA UTs are served by an AP, where one UT is near and the other UT is far away from the AP, and the far UT listens to the signal relayed by the FDR. There are three beamformer vectors to be optimized in this CNOMA channel, which are the AP transmit beamfomer, the FDR receive beamformer and the FDR transmit beamformer. The FD operation generates the SI at the relay receive antennas while the DaF protocol combined with CNOMA transmission makes the weakest link among the three links between the AP, FDR, and UTs determine the end-to-end throughput. These challenging features require novel idea and approaches to tackle this optimization problem. We propose two sub-optimal optimization algorithms for the design of the three aforementioned beamformers; the first one is the main algorithm while the second one is a benchmark scheme, which relies on the zero-forcing of the SI and the interference at the near UT. These beamformer optimization algorithms work on a metric that reflects the end-to-end sum rate (SR) of the CNOMA channel and is derived from mutual information of information theory (When the metric is based on mutual information, it is assumed that the transmission system applies a capacity approaching coding and modulation schemes for its physical layer. For example, the well-known combination of the low-density parity check coding scheme and quadrature amplitude modulation scheme may be adopted by the system). The main algorithm iterates the well-known quadratically constrained quadratic problem (QCQP) with the interference level at the near UT as a constraint in a while loop, whose exit conditions are related to the weakest link among the three links discussed above. Depending on the weakest link, the QCQP behaves differently against the interference level at the near UT, and the exit conditions of the while loop reflect these characteristics. The simulation results are presented to show the conditions, where the proposed algorithm outperforms the zero-forcing-based benchmark scheme and how the benefit of multiple antennas appears.

When malicious users overhear the legal transmissions without authorization, a security attack takes place, and the secure communication under such hazard eavesdropping is the main challenge of the physical layer security (PLS). The PLS has drawn research attraction recently [29], and the PLS in the NOMA channel or in the CNOMA channel has been considered in [20,21,30] as well when there are illegal eavesdroppers in the network. Since the well-known weapon for this PLS problem is beamforming through multiple antennas, we are interested in the modifications of the proposed beamforming algorithms for the CNOMA so that they can perform and improve the secure transmission under security attack as well. Here, the secrecy sum rate (SSR) is the optimization metric, and the SSR is also derived from mutual information. Note that the interference from the FDR degrades the reception of both the nearby UT and the eavesdropper, and this interference can be strategically exploited to enhance security. We propose a main algorithm leveraging this observation by modifying the proposed algorithm for the CNOMA channel slightly along with a modified zero-forcing algorithm. Simulation results showing the conditions where the modified algorithms behave differently and the impact of multiple antennas are presented.

In the numerical experiments, these two beamforming schemes are compared in terms of their performance across three different environments. The AP power, the FDR power and the number of antennas at the FDR are used for the experiment parameters to discuss out the pros and cons of proposed schemes. In Section 2, we present the system model of the proposed CNOMA with FDR. In Section 3, two beamforming optimization algorithms for the CNOMA channel are explained, and their performance comparison through simulations is presented. In Section 4, modifications of two algorithms for the security attacked CNOMA channel are discussed along with numerical results. Finally, Section 5 concludes this paper.

*Notations:* The notations $A^{H}$ and $A^{T}$ are the Hermitian transpose and the transpose of a matrix A, respectively. Tr[A] takes the trace of the matrix A, and rank{A} returns the rank of the matrix A. ∥a∥ denotes the $\ell_{2}$-norm of a vector a. The notation $A_{i,j}$ represents the element at the *i*-th row and the *j*-th column of A. The notation diag[v] produces a diagonal matrix with the elements of the vector v on its diagonal. The vector notations $0_{k}$ and $1_{j}$ represent all zero vectors with *k* elements and all one vector with *j* elements, respectively. CN(0,C) denotes the complex white Gaussian distribution of the random vector with zero mean vector 0 and the covariance matrix C. $C^{N}$ denotes the *N*-dimensional complex vector space. Finally, $E_{n}\left[x\right]$ takes the expectation of *x* with respect to *n*.

## 2. System Model

Consider the cooperative NOMA system where a multiple antenna AP transmits messages to 2 NOMA user terminals with a single antenna, respectively, where an FDR with multiple antennas delivers the message destined to the far user ($U_{2}$) whose direct channel from the AP is cut off by heavy blockages unlike the near user $U_{1}$ case. In this setting, Eve overhears the CNOMA transmission without authorization, and thus we try to maximize the CNOMA transmission rate, while the message is safe from the eavesdropping. The AP uses an M×1 AP beamformer vector $w_{A}$, where *M* denotes the number of transmit antennas at the AP with $∥w_{A}{∥}^{2}=P_{s}$ while the cooperative FDR has $N_{r}$ receive antennas and $N_{t}$ transmit antennas. The decode-and-forward (DaF) protocol-based FDR applies an $N_{r}\times 1$ unit-norm receive beamforming vector $w_{R}$ to the received signal to decode the messages from the AP. If the cooperative FDR decodes the AP messages for the two users, the signal for the second user is re-encoded before being beamformed by the $N_{t}\times 1$ transmit vector $w_{T}$ with $∥w_{T}{∥}^{2}=P_{r}$.

The multiple-input multiple-output (MIMO) channel from the AP to the cooperative FDR is denoted by the $N_{r}\times M$ matrix $H_{r}$ and the multiple-input single-output (MISO) channel from the AP to the 1-st user $U_{1}$ is denoted by the 1×M vector $h_{1}$ and the 1×M MISO channel vector from the AP to Eve is denoted as $h_{e}$. Also, the MIMO feedback channel from the transmit antennas to the receive antennas of the FDR is denoted by a $N_{r}\times N_{t}$ matrix $G_{RR}$, which is modeled as a rank-one matrix $G_{RR}=uv^{H}$, and the self-interference (SI) impacts the FDR reception circuit through this channel. Here, the vectors u and v are random unit norm vectors of dimensions $N_{r}\times 1$ and $N_{t}\times 1$, respectively. The $1\times N_{t}$ channel vector from the FDR to $U_{i},i=1,2$ is denoted as $g_{i},i=1,2$ and the $1\times N_{t}$ channel vector from the FDR to Eve is denoted as $g_{e}$. Note that all the elements of $H_{r}$, $h_{i},i=1,e$ and $g_{j},j=1,2,e$ are independent and identically distributed random variables with CN(0,1). We assume that full channel state information (CSI) is available at the AP and at the cooperative FDR via a channel learning process so that all the beamformer design is conducted at the AP or at the FDR (Note that well-known channel acquisition processes can be adopted for this CNOMA with the FDR channel, though the inclusion of the FDR and associated channels from it doubles the required resources for the channel learning. Both the AP and the FDR need to send separate pilot signals, while the user terminals and Eve should report both channel vector information. Note also that Eve is assumed to be served by the AP and is reporting its channel information to the network so that the network can utilize this information to protect other secure transmissions such as the CNOMA transmission of this work).

We assume that all the system nodes (AP, FDR, $U_{1}$, $U_{2}$, and Eve) are synchronized in time and frequency so that the received signals at the cooperative FDR, at $U_{1}$, at Eve, and at $U_{2}$ at time slot *n* are expressed, respectively, as

(1) $$\begin{array}{c} y_{r}\left(n\right)=H_{r}w_{A}x_{S}\left(n\right)+\sqrt{\alpha_{R}}G_{rr}w_{T}x_{r}\left(n-1\right)+n_{r}\left(n\right),\\ y_{k}\left(n\right)=h_{k}w_{A}x_{S}\left(n\right)+\sqrt{\alpha_{U}}g_{k}w_{T}x_{r}\left(n-1\right)+n_{k}\left(n\right),\\ k=1,e,\\ y_{2}\left(n\right)=\sqrt{\alpha_{D}}g_{2}w_{T}x_{r}\left(n-1\right)+n_{2}\left(n\right), \end{array}$$

where $x_{S}\left(n\right)=\sum_{k=1}^{2}x_{k}\left(n\right)$ is the composite AP message symbol with $E\left[|x_{S}{\left(n\right)|}^{2}\right]=1$ (Here, we assume that frame-based decoding and 1 unit delay at the FDR signal corresponds to one frame delay). The message $x_{k}\left(n\right)$ is destined to the *k*-th user with $E\left[|x_{k}{\left(n\right)|}^{2}\right]=\rho_{k}$ with $\rho_{1}+\rho_{2}=1$. The cooperative FDR applies the receive beamforming to the signal in (1) as $w_{R}^{H}y_{r}\left(n\right)$, decodes 2 messages with SIC manner from $x_{2}\left(n\right)$ to $x_{1}\left(n\right)$, re-encodes the far user message signal $x_{r}\left(n\right)=x_{2}\left(n\right)/\sqrt{\rho_{2}}$ and transmits with beamforming as $w_{T}x_{r}\left(n\right)$ at the time slot n+1. The vector $n_{r}\left(n\right)$ denotes an $N_{r}\times 1$ additive noise vector at the cooperative FDR receive antennas with $\mathrm{CN}(0,\sigma^{2}I_{N_{r}})$ distribution, while $n_{k}\left(n\right),k=1,2,e$ denotes the additive noise at the *k*-th UT or at Eve’s antenna with $\mathrm{CN}(0,\sigma^{2})$ distribution. It is assumed that the SI suppression at the FDR is composed of a two-step approach with analogue interference subtraction followed by digital beamforming discussed in this work to cope with the huge SI of FDR such that the interference level at the digital beamformer considered in this work is reduced within the dynamic range of the digital circuit. However, the SI level after the analogue subtraction still remains quite high, which leads to the introduction of the parameter $\alpha_{R},\alpha_{U}$ and $\alpha_{D}$ in (1) to emulate the situation where the self-interference at the FDR is dominating the signal from the source. The FDR transmit signal is scaled differently at the reaching nodes by controlling these parameters appropriately. The value $\alpha_{R}$ captures the capability of the analogue interference subtraction circuit, and the implementation details of the circuit [31] can be hidden, which allows the proposed beamforming schemes of this paper to concentrate only on the subtraction of the remaining SI in the digital domain.

The near user $U_{1}$, Eve, and the FDR use the observations from the time slot *n* in (1) to decode the two messages with SIC in a manner such that they decode the far user message $x_{2}\left(n\right)$ first, subtract out the related signal term, and decode the near user message signal $x_{1}\left(n\right)$. Note that the FDR signal is confusing Eve and $U_{1}$, as it is interfering their receptions of AP signal (Note that Eve and $U_{1}$ may try to decode the messages from the signal FDR sends as $U_{2}$ does, which requires us to use SINR expressions different from the ones in (2). However, the FDR signal does not contain $x_{1}\left(n\right)$, so we assume, here, that Eve and $U_{1}$ decode the two user messages from the signal AP sent and the SINR expressions in (2) are valid). For these, the signal-to-interference-plus-noise ratios (SINRs) for the signals $x_{j}\left(n\right),\;j=1,2$ are as follows:

(2) $$\begin{array}{cc} \gamma_{j}^{r} & =\frac{|w_{R}^{H}H_{r}w_{A}{|}^{2}\rho_{j}}{\alpha_{R}|w_{R}^{H}G_{rr}w_{T}{|}^{2}+{\left|w_{R}^{H}H_{r}w_{A}\right|}^{2}\sum_{m=1}^{j-1}\rho_{m}+\sigma^{2}},\\ \gamma_{j}^{k} & =\frac{|h_{k}w_{A}{|}^{2}\rho_{j}}{\alpha_{U}|g_{k}w_{T}{|}^{2}+{\left|h_{k}w_{A}\right|}^{2}\sum_{m=1}^{j-1}\rho_{m}+\sigma^{2}},\;k=1,e. \end{array}$$

Meanwhile, the far user $U_{2}$ decodes the signal $x_{2}\left(n\right)$ from the observation in (1) with the SINR given as

(3) $$\begin{array}{cc} \gamma_{2}^{2} & =\frac{\alpha_{D}{\left|g_{2}w_{T}\right|}^{2}}{\sigma^{2}}. \end{array}$$

With the DaF protocol at the FDR, the throughput rate (the information theoretic mutual information) of the near user signal $x_{1}\left(n\right)$ is given as $\log(1+\min\left[\gamma_{1}^{r},\gamma_{1}^{1}\right])$, while the throughput rate of the far user signal $x_{2}\left(n\right)$ is $\log(1+\min\left[\gamma_{2}^{r},\gamma_{2}^{1},\gamma_{2}^{2}\right])$. The throughput rates of these signals at Eve are given as $\log(1+\gamma_{1}^{e})$ and $\log(1+\gamma_{2}^{e})$, respectively. Hence, the SSR of the systems with two conditions $\min\left[\gamma_{1}^{r},\gamma_{1}^{1}\right]\geq \gamma_{1}^{e}$ and $\min\left[\gamma_{2}^{r},\gamma_{2}^{1},\gamma_{2}^{2}\right]\geq \gamma_{2}^{e}$ is given as

(4) $$\begin{array}{cc} R_{SSR} & =\log\left(1+\min\left[\gamma_{1}^{r},\gamma_{1}^{1}\right]\right)-\log\left(1+\gamma_{1}^{e}\right)\\ & \;\;+\log\left(1+\min\left[\gamma_{2}^{r},\gamma_{2}^{1},\gamma_{2}^{2}\right]\right)-\log\left(1+\gamma_{2}^{e}\right)\\ & =\log\left(\frac{\left(1+\min\left[\gamma_{1}^{r},\gamma_{1}^{1}\right]\right)\left(1+\min\left[\gamma_{2}^{r},\gamma_{2}^{1},\gamma_{2}^{2}\right]\right)}{\left(1+\gamma_{1}^{e}\right)\left(1+\gamma_{2}^{e}\right)}\right). \end{array}$$

## 3. Beamformer Design for the CNOMA with FDR and No Eve

**Figure 1:**
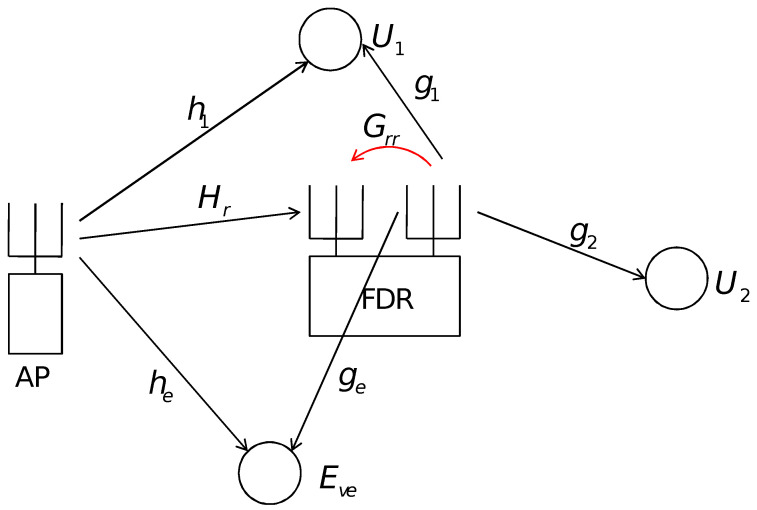
Cooperative Non-Orthogonal Multiple Access Transmission system through an FDR with 2 user terminals under security attack from the eavesdropper (Eve). The FDR operates in the DaF protocol. The AP and the FDR have multiple antennas while the two user terminals and Eve are equipped with single antennas, respectively. The direct channel from the AP to the far user ($U_{2}$) is blocked unlike the near user ($U_{1}$) case.

Here in this section, we assume that the Eve and associated channel vectors $h_{e}$ and $g_{e}$ disappear from Figure 1 and thus the system is free from security attacks. Then, the system model becomes plain cooperative NOMA (CNOMA) channel and we need to design the beamformer set $w_{A}$ at AP and $w_{T}$ with $w_{R}$ at FDR such that the FDR and UTs can decode their signals with the SIC manner at the maximum rate (The user terminals and Eve may have multiple antennas, and their receive beamforming can pose challenges. However, the CNOMA scheme in this paper assumes single stream transmission, which eases such challenges since the optimal beamforming for this case is well known to be eigen-based beamforming. The UT1 should consider the two matrix channels from the AP and from the FDR to form generalized eigen beamforming while the Eve and the UT2, respectively, should consider the matrix channel either from the AP or from the FDR to form the eigen beamforming). The optimal design of beamformers is complicated even in this plain CNOMA channel since the signals from the AP and FDR affect $U_{1}$ at the same time. We are interested in the sum rate of CNOMA denoted as $R_{SR}$, which is calculated by setting $\gamma_{i}^{e},\;i=1,2$ to zeroes. The minimum operators in the $R_{SSR}$ in (4) due to the DaF protocol make the problem even worse. We provide two beamforming schemes, where the first one relies on the iterative application of convex optimizations and the zero-forcing method until a convergent condition is met. The other scheme performs the benchmark role to be compared to the first one in performance and it relies purely on the zero-forcing method.

### 3.1. Design Based on QCQP

Since Eve disappears from Figure 1 and no security concern exists in the system, the SSR in (4) is reduced to the simple CNOMA sum rate (SR) as

(5) $$R_{SR}=\log\left(1+\min\left[\gamma_{1}^{r},\gamma_{1}^{1}\right]\right)+\log\left(1+\min\left[\gamma_{2}^{r},\gamma_{2}^{1},\gamma_{2}^{2}\right]\right).$$

It is not hard to see that maximizing $\min[\gamma_{1}^{r},\gamma_{1}^{1}]$ can be absorbed into maximizing $\min[\gamma_{2}^{r},\gamma_{2}^{1},\gamma_{2}^{2}]$, which maximizes $R_{SR}$ as a result. Recall the well-known fact that the maximum of $\min[\gamma_{2}^{r},\gamma_{2}^{1},\gamma_{2}^{2}]$ is achieved at the point with $\gamma_{2}^{r}=\gamma_{2}^{1}=\gamma_{2}^{2}$. Here, the transmission from the FDR toward $U_{2}$ interferes with the receptions at $U_{1}$ and at the FDR itself, which is reflected in the SINR expressions in (2). Handling this cross-interference (CR) and self-interference (SI) should be taken into consideration in the following beamformer design problem maximizing the CNOMA SR as

(6) $$P_{1}:\;\;\max_{w_{A},w_{R},w_{T}}\;\min\left[\gamma_{2}^{r},\gamma_{2}^{1},\gamma_{2}^{2}\right]).$$

For convenience in the subsequent development, we set the FDR reception beamformer as $|w_{R}^{H}G_{rr}w_{T}|=0$, i.e., the reception vector $w_{R}$ zero-forces the SI, which is reasonable and relevant since the SI term in (2) is dominating in the denominator of $\gamma_{j}^{r}$ as $\alpha_{R}|w_{R}^{H}G_{rr}w_{T}{|}^{2}\gg {\left|w_{R}^{H}H_{r}w_{A}\right|}^{2}\sum_{m=1}^{j-1}\rho_{m}$ for j=1,2. This condition relies on the assumption that the FDR transmit beamformer $w_{T}$ is determined already. Further, we set the AP beamfomer $w_{A}$ such that the AP transmission arrives at $U_{1}$ and FDR with almost equal power, i.e., we set $w_{A}$ such that the term $∥|w_{R}^{H}H_{r}w_{A}\left|-\right|h_{k}w_{A}|∥$ is minimized with $∥w_{A}{∥}^{2}=P_{s}$. Again, this condition implies that the FDR reception beamformer $w_{R}$ is determined already. Consequently, these two conditions require a chain of beamformer decision sequence as $w_{T}\to \;w_{R}\to \;w_{A}$. Later in this subsection, we develop an iteration-based approach, where the determination of beamformers follows the former sequence in a cyclic manner. This cyclic manner can be implemented by introducing a $w_{T}$ determination method with $w_{A}$ and $w_{R}$ fixed, which requires some modifications of $P_{1}$ in (6) with the second NOMA user rate as

(7) $$\begin{array}{cc} {\tilde{P}}_{1}:\;\;\max_{w_{T}}\; & R_{2}=\min\left[{\tilde{\gamma }}_{2}^{r},\gamma_{2}^{1},\gamma_{2}^{2}\right])\\ \; & {\tilde{\gamma }}_{2}^{r}=\frac{|w_{R}^{H}H_{r}w_{A}{|}^{2}\rho_{2}}{|w_{R}^{H}H_{r}w_{A}{|}^{2}\rho_{1}+1}. \end{array}$$

Note that the FDR transmit beamformer $w_{T}$ indirectly affects ${\tilde{\gamma }}_{2}^{r}$ through the condition $|w_{R}^{H}G_{rr}w_{T}|=0$, though it does not appear in (7).

The design of the optimization algorithm for the beamforming vector set relies on the characteristics given by the minimum operator in (6) and in (7). Due to the minimum operator, the rate $R_{2}$ is dominated by the weakest node signal quality among the three nodes of the FDR, $U_{1}$, and $U_{2}$. This fact can be exploited in conjunction with the following formation of popular convex optimization technique. With the matrix definitions ${\bar{G}}_{k}=g_{k}g_{k}^{H}$ for k=1,2, and ${\bar{G}}_{r}=G_{rr}^{H}w_{R}w_{R}^{H}G_{rr}$, we form the following QCQP (quadratically constrained quadratic problem):

(8) $$\begin{array}{cc} \max_{w_{T}} & |w_{T}^{H}\bar{G_{2}}w_{T}|\\ \;s.t. & {∥w_{T}∥}^{2}=P_{r},\\ \; & |w_{T}^{H}\bar{G_{1}}w_{T}|\leq \delta_{1}. \end{array}$$

Here, $\delta_{1}$ controls the cross-interference (CI) level at $U_{1}$ while the maximization is to strengthen the channel toward $U_{2}$ (Note that in the case where $U_{1}$ disappears and the FDR performs the near user role from Figure 1, then Algorithm 1 does not work at all since it relies heavily on $\delta_{1}$). This formation of QCQP is versatile in building the central block of Algorithm 1 since it provides a trade-off among two SINRs at $U_{1}$ and $U_{2}$. Note that the while loop in Algorithm 1 updates the beamformer vector set $w_{A},w_{R}$ and $w_{T}$ in the cyclic sequence given above iteratively. Meanwhile, the range of CI level $\delta_{1}$ in (8) is tested with a four-footed rake-type selection of the CI level points. The result of this test determines if the while loop terminates or continues with a halved CI level range.

The four test CI levels are determined such that two of them are near the two end points (1 % and 99 % of the range) of the CI level test range while the other two levels are placed at 25 % and at 75 % points of the range, respectively. If the maximum $R_{SR}$ value among those four values at the test points is achieved at the point near a far end, the while loop is terminated with $\delta_{old}$ set to the near end value. Otherwise, the loop continues with the CI level range, which is reduced to the half range containing the test point with the maximum $R_{SR}$ value. The while loop keeps on until the maximum $R_{SR}$ does not increase more than a marginal value. After the while loop, the algorithm performs the optimization of beamfomring vector set again with the $\delta_{old}$, the middle value of the last CI level range. Note that the strategy behind Algorithm 1 is carefully crafted to utilize the fact that the weakest signal node among the three nodes dominates the CNOMA SR $R_{2}$. This again makes $R_{2}$ depend on the CI level $\delta_{1}$ in four types. In the first type, $R_{2}$ monotonically increases with $\delta_{1}$ when the SINR at $U_{2}$ is the weakest, and maximizing the link to $U_{2}$ with (8) is achieved by setting $\delta_{1}$ to be the largest value. In the second type, $R_{2}$ monotonically decreases with $\delta_{1}$ when the SINR at $U_{1}$ is the weakest, and maximizing the SINR is achieved by setting $\delta_{1}$ to be the smallest value. The third type happens when the weakest SINR occurs at $U_{2}$ with small $\delta_{1}$ and the one at $U_{1}$ becomes the weakest as δ increases, which makes $R_{2}$ a concave function of $\delta_{1}$. Finally, $R_{2}$ is not affected by $\delta_{1}$ when the SINR at FDR is the weakest. The while loop iteratively reduces the $\delta_{1}$ test range to capture the peak of the concave function in the third case, and the loop is terminated with $\delta_{1}$ set to the end value of the range when the first two cases occur. Finally, note that the computational complexity depends mostly on the QCQP and the associated one-dimensional search over $\delta_{1}$ in the while loop (Compared to the successive convex approximation (SCA) approach as in [32,33,34], Algorithm 1 applies two different convex optimizations iteratively in the while loop, where one-dimensional search is used for $w_{A}$ and the QCQP for $w_{T}$).
**Algorithm 1** The iterative optimization algorithm for the CNOMA with FDR1:Set $SR_{old}=0$.2:Set $\delta_{1,min}=0$, $\delta_{1,max}=P_{r}{∥g_{1}∥}^{2}$.3:Set $w_{T}={\left(\frac{P_{g_{1}}\left(g_{2}\right)}{∥P_{g_{1}}\left(g_{2}\right)∥}\right)}^{H}$ and $w_{R}^{*}=\frac{P_{G_{\mathrm{rr}}w_{T}}\left(e_{1}\right)}{∥P_{G_{\mathrm{rr}}w_{T}}\left(e_{1}\right)∥}$. Here, $e_{1}$ is the left singular vector of $H_{r}$ with the largest singular value. Set $w_{A}$ such that $∥|w_{R}^{H}H_{r}w_{A}\left|-\right|h_{1}w_{A}|∥$ is minimized with $∥w_{A}{∥}^{2}=P_{s}$ through a one-dimensional search.4:**while** ( ) **do**5:    Set $\delta_{1}=\left(\delta_{1,min}+\delta_{1,max}\right)/2$ and $\Delta_{1}=\delta_{1,max}-\delta_{1,min}$.6:    Calculate four thresholds $\tau_{1}=\delta_{1,min}+0.01\Delta_{1},\tau_{2}=\delta_{1,min}+\Delta_{1}/4,\tau_{3}=\delta_{1,max}-\Delta_{1}/4,\tau_{4}=\delta_{1,max}-0.01\Delta_{1}$.7:    Repeat the optimization in (8) with four τ’s calculated above for $\delta_{1}$ values. For the found four $w_{T}$’s, renew $w_{R}$ and $w_{A}$ as in item 3 and calculate $R_{SR}=\log\left(1+\min\left[\gamma_{1}^{r},\gamma_{1}^{1}\right]\right)+\log\left(1+\min\left[\gamma_{2}^{r},\gamma_{2}^{1},\gamma_{2}^{2}\right]\right)$ using (2) and (3). Set the calculated SR values as $SR_{i}$’s, where $SR_{i}$ is the calculated SR value with $\tau_{i}$. Choose the maximum SR value as $SR_{max}=\max\left\{SR_{1},SR_{2},SR_{3},SR_{4}\right\}$.8:    **if** ($SR_{max}=SR_{4}$ or $SR_{max}=SR_{1}$) **then**9:         Set $\delta_{1,old}=\delta_{1,max}$ if $SR_{4}$ is the maximum and $\delta_{1,old}=\delta_{1,min}$ otherwise. Break the while loop.10:    **else**11:         $\delta_{1,max}=\delta_{1}$ if $SR_{2}$ is the maximum and $\delta_{1,min}=\delta_{1}$ otherwise.12:    **end if**13:    **if** ($SR_{max}-SR_{old}\leq 0.001$) **then**14:         Break the while loop.15:    **else**16:         $SR_{old}=SR_{max}$. and $\delta_{old}=\delta_{1}$.17:    **end if**18:**end while**19:Perform the optimization in (8) with $\delta_{old}$ for $\delta_{1}$ value. For the new $w_{T}$, find $w_{R}$ and $w_{A}$ as in item 320:Calculate the final $R_{SR}$ using (2) and (3).

### 3.2. Zero-Forcing for the CNOMA with FDR

Algorithm 1 has iterative applications of QCQP with one-dimensional search over parameter $\delta_{1}$, and thus it requires quite amount of computational load. Here, we propose a simplified scheme in Algorithm 2, which zero-forces the SI as Algorithm 1 while the interference from the FDR to $U_{1}$ is also zero-forced. The SI is suppressed by the FDR receive beamfomer ($w_{R}$) and the interference at $U_{1}$ is suppressed by the FDR transmit beamformer ($w_{T}$). The AP beamformer is found by the same method as in Algorithm 1. This scheme considers the second hop (FDR to NOMA user channel) as if only a single user is served so that the QCQP is not needed.
**Algorithm 2** The zero-forcing-based algorithm for CNOMA1:Set $w_{T}={\left(\frac{P_{g_{1}}\left(g_{2}\right)}{∥P_{g_{1}}\left(g_{2}\right)∥}\right)}^{H}$ and $w_{R}^{*}=\frac{P_{G_{\mathrm{rr}}w_{T}}\left(e_{1}\right)}{∥P_{G_{\mathrm{rr}}w_{T}}\left(e_{1}\right)∥}$. Here, $e_{1}$ is the left singular vector of $H_{r}$ with the largest singular value. Find $w_{A}$ such that $∥|w_{R}^{H}H_{r}w_{A}\left|-\right|h_{1}w_{A}|∥$ is minimized with $∥w_{A}{∥}^{2}=P_{s}$ through a one-dimensional search.2:Scale $w_{T}$ such that $∥w_{T}{∥}^{2}=P_{r}$.3:Calculate $R_{SR}$ using (2) and (3).

### 3.3. Numerical Results 1

**Figure 2:**
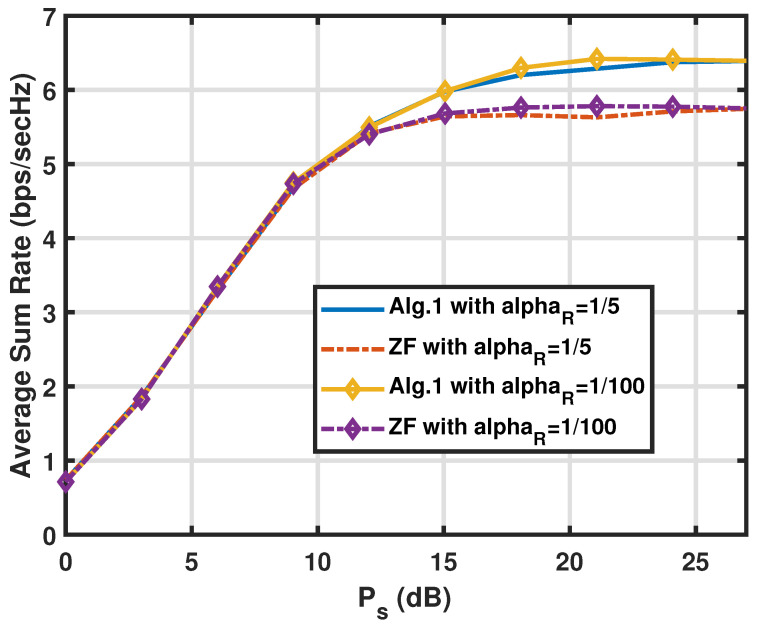
Comparison of the sum rate $R_{SR}$ of the proposed algorithms for the FDR CNOMA channel against the source transmit power with different $\alpha_{R}$ values when $M=N_{r}=N_{t}=3,P_{r}=42\;\left(\mathrm{dB}\right)$, and $\alpha_{R}=1/5$ or $\alpha_{R}=10^{-2}$. The path-loss from the FDR to users $\alpha_{U}$ is set to 1/100 to scale the big $P_{r}$ values down to realistic levels at users since the $P_{r}$ values are taken to reflect strong SI power after the analogue SI cancellation.

In this subsection, we analyze the average sum rate ($R_{SR}$) performance of the proposed CNOMA transmission with the FDR mode of Algorithm 1 and Algorithm 2, respectively, in different parameter configurations. Here, we have two users ($U_{1}$, $U_{2}$), two values of path-gain of the SI link as $\alpha_{R}=10^{-2}$ or $\alpha_{R}=1/5$ and $\rho_{k}=1/2\;\mathrm{for}\;k=1,2$ when the analogue SIC circuit at the FDR removes a considerable amount of SI. The specific parameter value settings for the FDR relaying scenario follow those in [35] with $\alpha_{D}=\alpha_{U}=1/100$. The assumption that all the elements of the channel vectors are *i.i.d* complex Gaussian variables implies the flat fading channel with Rayleigh distribution. The sum rates of the two algorithms are presented in Figure 2 against the source transmit power $P_{s}$ (first-hop link) with two different $\alpha_{R}$ values (i.e., different SI link conditions) and the FDR power $P_{r}=42\left(dB\right)$ for the CNOMA FDR case. Overall, the comparison of the curves shows that the Algorithm 1 does not perform better than the ZF scheme (in Algorithm 2) until $P_{s}$ increases over 10(dB). This implies that the ZF-based scheme is enough in the regime with $P_{s}$ being less than 10(dB) since the SINRs at $U_{1}$ and FDR are weaker than the one at $U_{2}$ in the regime. Also, note that with the variation of the $\alpha_{R}$ value, it does not make a noticeable difference to say that the remaining SI after the analogue SIC is effectively removed by both algorithms and that the spectrally efficient merit from the full duplex operation is collected without much toll.

**Figure 3:**
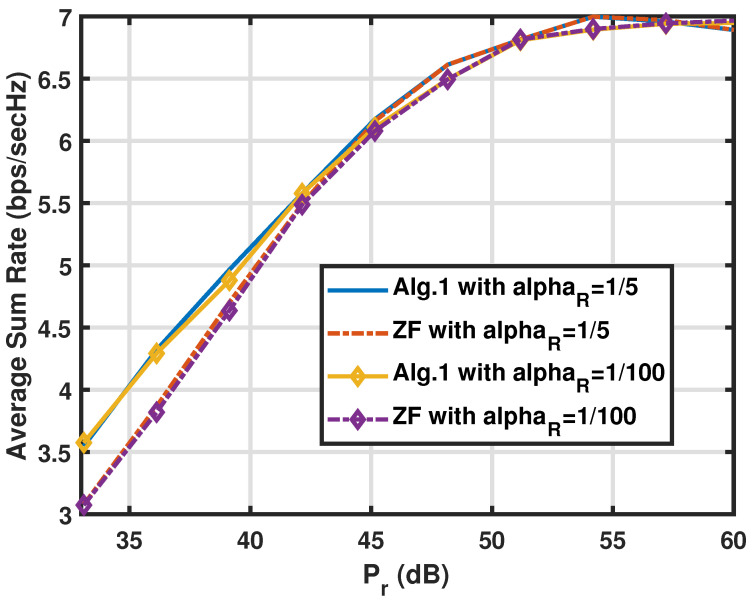
Comparison of the sum rate $R_{SR}$ of the proposed algorithms for the FDR CNOMA channel against the FDR transmit power with different $\alpha_{R}$ values when $M=N_{r}=N_{t}=3,P_{s}=15\;\left(\mathrm{dB}\right)$, and $\alpha_{R}=1/5$ or $\alpha_{R}=10^{-2}$. The path-loss from the FDR to users $\alpha_{U}$ is set to 1/100.

**Figure 4:**
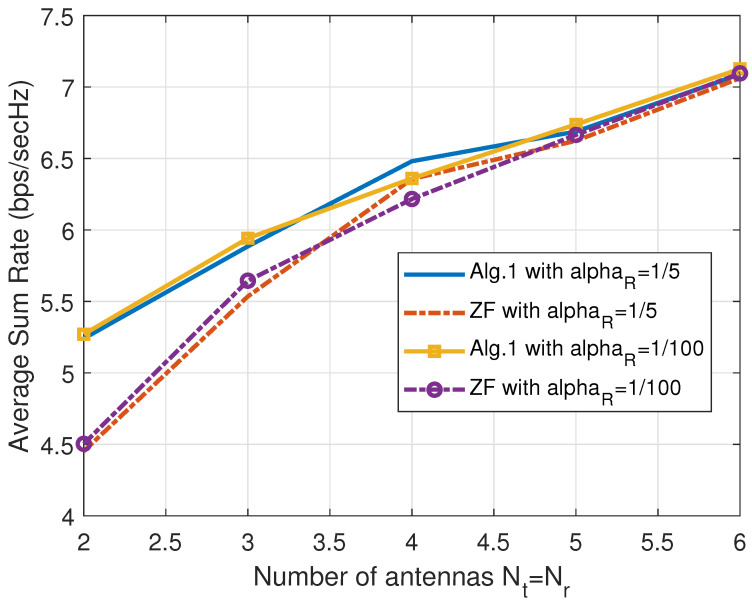
Comparison of the sum rate $R_{SR}$ of the proposed algorithms for the FDR CNOMA channel against the number of FDR transmit antennas $N_{t}=N_{r}$ with different $\alpha_{R}$ values when $M=3,\;P_{r}=42\;\left(\mathrm{dB}\right),P_{s}=15\;\left(\mathrm{dB}\right)$, and $\alpha_{R}=1/5$ or $\alpha_{R}=10^{-2}$. The path-loss from the FDR to users $\alpha_{U}$ is set to 1/100. Here, only the points of the integer numbers of antennas are valid ones (simulated ones), though we interpolate those points to make curves.

Contrasting the simulations in Figure 2, the experiments in Figure 3 exhibit the average sum rates $R_{SR}$ of the two algorithms against the FDR power $P_{r}$ with two different values of $\alpha_{R}$. Here, the gain of Algorithm 1 diminishes as the $P_{r}$ values grow, which can be explained if we refer to the other side of the lesson we learned from the experiments in Figure 2. In other words, as the first-hop SINRs become the weaker ones among the node SINRs, the ZF-based scheme of Algorithm 2 is good enough to harvest the CNOMA throughput. Again, the $\alpha_{R}$ value does not make a big difference in the sum rates. Note that the smaller the $\alpha_{R}$ value is, the better the SI is suppressed by the analogue SI subtraction circuit. Both systems exploit the multiple transmit and receive antenna sets ($N_{t}$ and $N_{r}$) at the FDR to harvest the gain in the CNOMA transmission. The throughput gain from large number of antennas diminishes as the number of antennas increases for both of the two algorithms. In Figure 4, we compare the sum rate performance of the proposed systems against the number of FDR antennas when we have $N_{t}=N_{r}$. Again, the ZF-based Algorithm 2 exhibits strong performance when the number of FDR antennas is substantial. Recall that the elements of channel vectors are assumed to be *i.i.d.* flat Rayleigh fading variables and there exists no consideration for the antenna configuration. However, such an assumption can be met with a linear antenna array with appropriate separation while the line-of-sight paths are heavily blocked.

## 4. Application of Algorithms to the Security Attack Scenario

Now, we assume that Eve is overhearing the AP transmission with the interference signal from the FDR as in Figure 1. Here, we need to consider $R_{SSR}$ as appeared in (4). A similar chain of reasoning to the one in Section 3 leads us to consider a simplified version of the secrecy rate over $R_{SSR}$, which can be given as

(9) $${\tilde{R}}_{SSR}=\log\left(1+\min\left[\gamma_{2}^{r},\gamma_{2}^{1},\gamma_{2}^{2}\right]\right)-\log\left(1+\gamma_{2}^{e}\right).$$

Note that the appearance of Eve requires us to take the AP to Eve link and the FDR to Eve link into consideration since they affect $\gamma_{1}^{e}$, the SINR at Eve. We, in this section, will show that the algorithms developed in the previous section can be applied for the SSR maximization with slight modifications.

### 4.1. Modifications of Algorithms in Section 3

In the configuration of this section, the first link (AP to Eve) becomes the source of overhearing, while the second link (FDR to Eve) performs the opposite role of hindering Eve from eavesdropping. Obviously, two strategies of suppressing the first link and strengthening the second link are helpful for the maximization of SSR in (4). Suppressing the first link can be incorporated in the zero-forcing scheme in Algorithm 3, where a slight modification of Algorithm 2 is made by projecting the found $w_{A}$ onto the orthogonal direction of $h_{e}$. It is natural to guess that the zero-forcing scheme has superior performance when the first link dominates over the second link, and it will be supported by the simulation results in Section 4.2 later. On the other hand, when the second link dominates the first link, strengthening the second link is a good approach, and it can be incorporated by modifying the QCQP in (8) as

(10) $$\begin{array}{cc} \max_{w_{T}} & |w_{T}^{H}\left(\bar{G_{2}}+\bar{G_{e}}\right)w_{T}|\\ \;s.t. & {∥w_{T}∥}^{2}=P_{r},\\ \; & |w_{T}^{H}\bar{G_{1}}w_{T}|\leq \delta_{1}. \end{array}$$

Here, ${\bar{G}}_{e}=g_{e}g_{e}^{H}$. Note that this modification of the objective function strengthens both the channel gains toward $U_{2}$ and Eve from the FDR and does not change the concave property of the problem. In this way, a part of the FDR signal toward $U_{2}$ is directed toward Eve so that the signal from AP toward Eve is interfered.

The above two modifications enable us to apply Algorithm 1 to the security attack scenario in Figure 1. The key point in applying these modifications is the way to decide which link among the two additional links in the security attack dominates over the other one. A set of simulations informs us that it is clever to compare the source power $P_{s}$ and the FDR power $P_{r}$ such that we declare (**case I**) the first link to be dominating over the second link when $2P_{s}>\alpha_{U}P_{r}$, and the opposite situation (**case II**) otherwise. In **case I**, every time the AP beamformer $w_{A}$ is updated, it is projected onto the orthogonal space of $h_{e}$ and normalized as in Algorithm 3. In this case, trying to hinder Eve from overhearing the AP signal by concentrating the interference from FDR is not much likely to work well and it is better, instead, to null out the AP signal toward Eve. For **case II**, we replace the optimization in Algorithm 1 from Equation (8) to Equation (10), while keeping all other components of the algorithm unchanged. Here, it is clever to interfere with the overhearing of the AP signal by Eve with the signal from FDR, which is achieved by the newly added term in the objective function in (10). Algorithm 3 performs the benchmark role again in this scenario.
**Algorithm 3** The zero-forcing beamforming for the SSR with FDR1:Set $w_{T}={\left(\frac{P_{g_{1}}\left(g_{2}\right)}{∥P_{g_{1}}\left(g_{2}\right)∥}\right)}^{H}$ and $w_{R}^{*}=\frac{P_{G_{\mathrm{rr}}w_{T}}\left(e_{1}\right)}{∥P_{G_{\mathrm{rr}}w_{T}}\left(e_{1}\right)∥}$. Here, $e_{1}$ is the left singular vector of $H_{r}$ with the largest singular value. Find ${\bar{w}}_{A}$ such that $∥|w_{R}^{H}H_{r}w_{A}\left|-\right|h_{1}w_{A}|∥$ is minimized with $∥w_{A}{∥}^{2}=P_{s}$ through a one-dimensional search. Form $w_{A}=P_{h_{e}}\left({\bar{w}}_{A}\right)/∥P_{h_{e}}\left({\bar{w}}_{A}\right)∥$.2:Scale $w_{T}$ such that $∥w_{T}{∥}^{2}=P_{r}$.3:Calculate $R_{SSR}$ using (4).

### 4.2. Numerical Results 2

In this subsection, we analyze the average secrecy sum rate ($R_{SSR}$) performance of Algorithm 1 modified as in previous Section 4.1 and of Algorithm 3 for the CNOMA transmission with the FDR mode and security attack from an eavesdropper in different parameter configurations. The same simulations of Section 3.3 are performed except that there exists an eavesdropper overhearing the CNOMA transmission.

**Figure 5:**
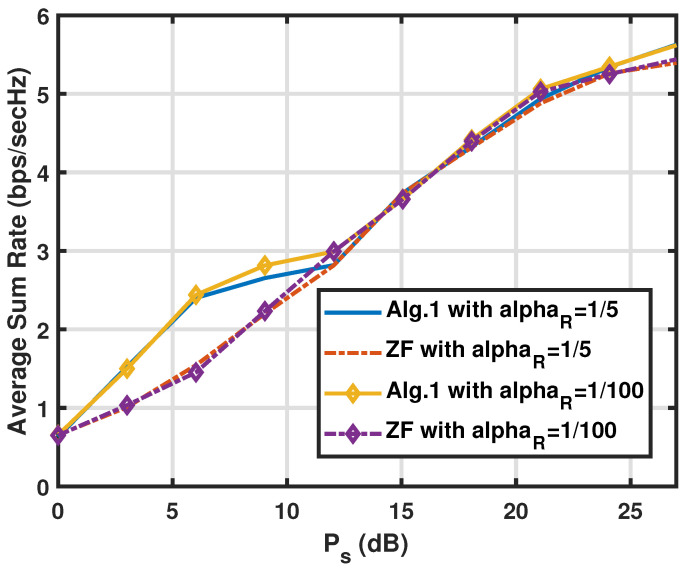
Comparison of the secrecy sum rate $R_{SSR}$ of the proposed algorithms for the FDR CNOMA eavesdropping channel against the source transmit power with different $\alpha_{R}$ values when $M=N_{r}=N_{t}=3,P_{r}=42\;\left(\mathrm{dB}\right)$, and $\alpha_{R}=1/5$ or $\alpha_{R}=10^{-2}$. The path-loss from the FDR to users $\alpha_{U}$ is set to 1/100 to scale the big $P_{r}$ values down to realistic levels at users since the $P_{r}$ values are taken to reflect strong SI power after the analogue SI cancellation.

**Figure 6:**
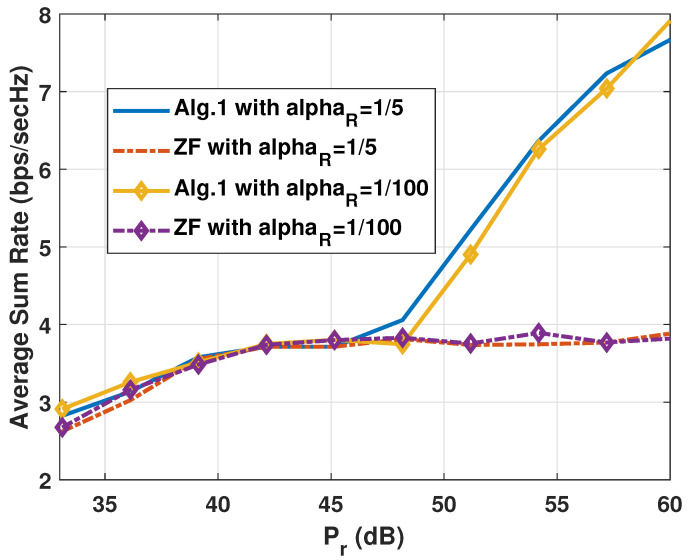
Comparison of the secrecy sum rate $R_{SSR}$ of the proposed algorithms for the FDR CNOMA eavesdropping channel against the FDR transmit power with different $\alpha_{R}$ values when $M=N_{r}=N_{t}=3,P_{s}=15\;\left(\mathrm{dB}\right)$, and $\alpha_{R}=1/5$ or $\alpha_{R}=10^{-2}$. The path-loss from the FDR to users $\alpha_{U}$ is set to 1/100.

**Figure 7:**
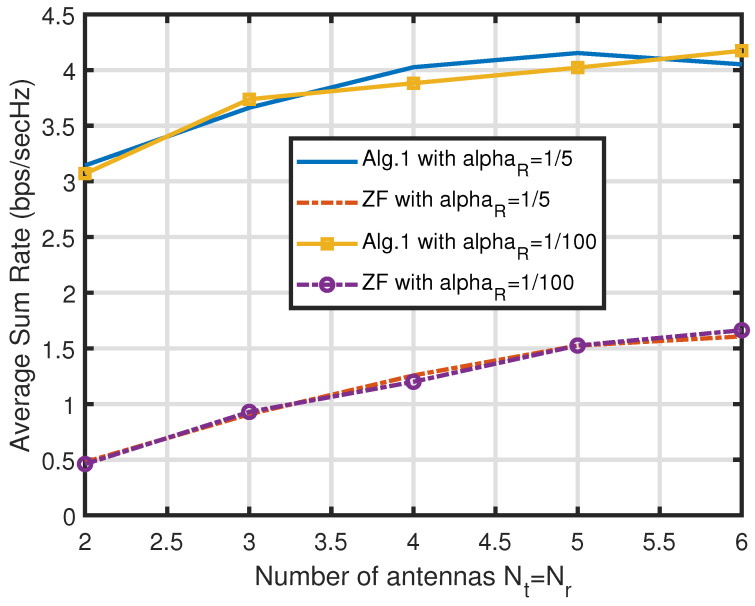
Comparison of the secrecy sum rate $R_{SSR}$ of the proposed algorithms for the FDR CNOMA eavesdropping channel against the number of FDR transmit antennas $N_{t}=N_{r}$ with different $\alpha_{R}$ values when $M=3,P_{r}=42\;\left(\mathrm{dB}\right),P_{s}=15\;\left(\mathrm{dB}\right)$, and $\alpha_{R}=1/5$ or $\alpha_{R}=10^{-2}$. The path-loss from the FDR to users $\alpha_{U}$ is set to 1/100. Here, only the points of the integer numbers of antennas are valid ones (simulated ones), though we interpolate those points to make curves.

Surprisingly, the curves in Figure 5 and in Figure 6 behave with drastically different trends compared to those we have seen in Figure 2 and in Figure 3, where the modified Algorithm 1 beats Algorithm 3 in the low $P_{s}$ regime in Figure 5 and in the high $P_{r}$ regime in Figure 6. However, this reversal in algorithm behavior aligns well with the intuition we developed when modifying Algorithm 1 in Section 4.1. Note that the condition we exploit for the modification is $2P_{s}\leq \alpha_{U}P_{r}$ (**case II**) and the other way around in (**case I**). The modification takes effect in this **case II** and shows superior performance to that of the zero-forcing-based Algorithm 3 while it fails to make a noticeable difference in **case I**. Note also that the performance comparison in Figure 7 against the numbers of FDR antennas $\left(N_{t}=N_{r}\right)$ again shows some difference from that in Figure 4. The performance gaps between the modified Algorithms 1 and 3 become wider and more persistent, even as the number of antennas increases. This implies that the beamforming gain for the security attack from Eve has wider room for the multiple antennas to exert its ability in securing the information transmission compared to the scenario where there exists no such security concern as in Figure 4.

## 5. Conclusions

Optimization schemes for the beamforming of the cooperative non-orthogonal multiple access (CNOMA) transmission with a full duplex relay (FDR) and Decode-and-Forward (DaF) protocol are proposed along with their application to the security attack scenario. The first proposed algorithm iteratively applies the quadratically constrained quadratic problem (QCQP) in a while loop with constraining the interference power level at the near user to exploit the characteristics that FDR and DaF impose for the CNOMA channel. As a reference scheme, a zero-forcing-based one is proposed for the FDR CNOMA channel. For the security attack case, the proposed schemes are presented with minor modifications. Surprisingly, the condition where the first algorithm outperforms the zero-forcing-based one is drastically different in the plain CNOMA channel and in the security attacked CNOMA channel such that the first algorithm beats the zero-forcing-based one when the AP power $P_{s}$ is strong enough in the plain CNOMA channel. On the other hand, the trend is reversed in the security-attacked CNOMA channel. This can be explained by the fact that the stronger the FDR transmit power $P_{r}$, the better the chance for the first algorithm to exploit this power to interfere with the Eve overhearing the AP transmission in the security attacked CNOMA. On the other hand, the FDR to the far user $U_{2}$ link becomes the weakest one, as the AP power grows in the plain CNOMA, and the first algorithm has more opportunities to make a trade-off for the improved throughput.

## Data Availability

All the data presented in the paper are generated by our own in-house simulations and they can be acquired if there exist any requests from readers. Please, feel free to contact us in case the presented data or the matlab codes for them are needed.
